# Brain white matter hyperintensities in Kawasaki disease: A case–control study

**DOI:** 10.3389/fnins.2022.995480

**Published:** 2022-10-18

**Authors:** Dan Laukka, Riitta Parkkola, Jussi Hirvonen, Pauli Ylikotila, Tero Vahlberg, Eeva Salo, Juri Kivelev, Jaakko Rinne, Melissa Rahi

**Affiliations:** ^1^Department of Neurosurgery, Neurocenter, Turku University Hospital, Turku, Finland; ^2^Clinical Neurosciences, University of Turku, Turku, Finland; ^3^Department of Radiology, Turku University Hospital, University of Turku, Turku, Finland; ^4^Neurocenter, Turku University Hospital, Turku, Finland; ^5^Department of Clinical Medicine, Biostatistics, University of Turku, Turku, Finland; ^6^Children's Hospital, Helsinki University Hospital, University of Helsinki, Helsinki, Finland

**Keywords:** Kawasaki disease, brain MRI, follow up, white matter hyperintensities, cerebrovascular abnormalities

## Abstract

**Background:**

Cerebrovascular involvement of Kawasaki disease (KD) is poorly studied. White matter hyperintensities (WMH) indicate cerebral small vessel disease and increase the risk for stroke.

**Purpose:**

To investigate whether childhood KD is associated with WMHs and other cerebrovascular findings later in adulthood.

**Materials and methods:**

In this case-control study, patients diagnosed with KD (cases) at our tertiary hospital between 1978 and 1995 were invited to brain magnetic resonance (MRI) between 2016 and 2017. Migraine patients (controls) with available brain MRI were matched with cases (ratio 4:1) by age (±2 years) and sex. Two blinded neuroradiologists evaluated independently cerebrovascular findings from the brain MRI scans. Modified Scheltens' visual rating scale was used to evaluate WMH burden and the total WMH volume was measured using manual segmentation.

**Results:**

Mean age [years, (SD)] at the time of brain MRI was 33.3 (3.8) and 32.8 (4.0) for cases (*n* = 40) and controls (*n* = 160), respectively (*P* = 0.53). Mean follow-up time for cases was 29.5 years (4.3). Total volume of WMHs (median) was 0.26 cm^3^ (IQR 0.34) for cases and 0.065 cm^3^ (IQR 0.075) for controls, *P* = 0.039. Cases had higher total WMH burden (*P* = 0.003), deep WMH burden (*P* = 0.003), and more periventricular WMHs (prevalence 7.5 vs. 0%, *P* = 0.008) than controls. Cases had greater risk of having total Scheltens' score ≥2 vs. < 2 (odds ratio, 6.88; 95% CI: 1.84–25.72, *P* = 0.0041) and ≥3 vs. < 3 (odds ratio, 22.71; 95% CI: 2.57–200.53, *P* = 0.0049). Diabetes type 1/type 2, hypertension, smoking status or hypercholesterolemia were not risk factors for WMH burden, *p* > 0.1. Myocarditis at the acute phase of KD increased the risk for periventricular WMHs (*P* < 0.05). Three cases (7.5%) and three controls (1.9%) had lacune of presumed vascular origin (*P* = 0.0096).

**Conclusion:**

History of KD could be associated with an increased WMH burden. More studies are needed to confirm our results.

## Introduction

Kawasaki disease (KD) is a childhood vasculitis affecting small and medium-sized arteries of the entire body (Cohen and Sundel, 2016) and 30% may present central nervous system symptoms (Tizard, 2005) and signs of intracranial vasculitis (Amano and Hazama, 1980). Symptomatic brain infarct is rare in KD patients and is limited to case reports (Wang et al., 2021).

White matter hyperintensities (WMH) usually indicate cerebral small vessel disease (Wardlaw et al., 2015). High WMH burden is associated with an increased risk of death, stroke, psychiatric disorders, and dementia (Au et al., 2006; Debette et al., 2019). Prevalence of WMH increases with age from 5% in healthy young adults (Hopkins et al., 2006) to over 60% in elderly (Lam et al., 2021). Compared to the general population, migraine patients have a higher risk for WMHs (Kruit et al., 2004; Palm-Meinders et al., 2012; Hamedani et al., 2013) with a prevalence of 11% in children (Eidlitz-Markus et al., 2013) and 39–44% in young adults without a significant differences between migraine subtypes (Dobrynina et al., 2021).

Although previous studies suggest that KD affects also cerebral vessels (Amano and Hazama, 1980; Ichiyama et al., 1998; Tizard, 2005; Hikita et al., 2011) this area is poorly studied and it is unknown if KD is associated with WMHs later in adulthood (Muneuchi et al., 2006). However, a recent study found that KD might increase the risk for hemorrhagic and ischemic stroke (Lin et al., 2022).

The objective of this study was to investigate if KD is associated with WMHs and other cerebrovascular findings in the long-term follow-up.

## Materials and methods

### Standard protocol approvals, registrations, and patient consents

This study was approved by the Ethics Committee of the Hospital District of Southwest Finland. Written informed consent was obtained from all cases in the study. Informed consent was not required for controls, because controls were included from a retrospective register. All methods were performed in accordance with STROBE guidelines and the Declaration of Helsinki.

### Study population

#### Cases

KD patients who were diagnosed and treated in the catchment area of the Turku University Hospital (population of 887,000 citizens) from 1978 to 1995 were identified retrospectively by using diagnostic codes (International Classification Code-9, 446.1; International Classification Code-10, M30.3). Diagnosis was confirmed from the patient records for each patient according to American Heart Association (AHA) 2004 diagnostic criteria for complete KD (Newburger et al., 2004). Patients with a current age of ≥25 years and a history of KD occurring in childhood were included in this study. Age criteria were based on the protocol for this cohort described in the earlier study (Laukka et al., 2019). Patients with current age < 25 years, Marfans syndrome, Ehler-Danlos syndrome type IV, polycystic kidney disease, or history of intracranial aneurysms or bleeding were excluded. Patients with a positive family history of intracranial aneurysms were also excluded.

There were 87 KD patients diagnosed between 1978 and 1995. Of 87 KD patients, 27 were excluded because of age < 25 years. Based on a review of patient records, none of the 87 KD patients had been diagnosed with ischemic or hemorrhagic stroke before beginning the study enrollment year 2016. An invitation letter was sent to 60 patients who met the inclusion criteria and 40 of them were willing to participate in the study, and 20 refused. Prior to brain magnetic resonance imaging (MRI), patients were interviewed for past medical history (hypertension, diabetes mellitus, hypertension, migraine, hyperlipidemia, depression, history of stroke, neurological symptoms), medication, smoking, alcohol consumption, and possible signs of heart or lung problems.

Of 40 cases, 37 had accurate information on which drug KD had been treated with and 37 patients had accurate information about echocardiographic data during the acute phase of KD. Of 37 patients 22 were treated with intravenous immunoglobulin and 15 patients with aspirin only.

This was a population-based study since all the patients were collected from our catchment area.

#### Controls

All patients who had undergone brain MRI for any reason (*n* = 39,993) between 2003 and 2020 in our tertiary hospital were reviewed to include migraine patients (controls). Of these patients, 1,062 had migraine diagnoses (International Classification Code-10, G43.0-G43.3) in patient records. Of 1,062 migraine patients, 68 were excluded because of intracranial tumor, history of acute brain infarction, sinus thrombosis, or subarachnoid- /intracerebral hemorrhage. None of the controls had a history of KD, other vasculitis, or brain diseases. From 994 migraine patients, controls were matched (four controls to one case) randomly by age (±2 years at the time of the brain MRI) and sex with cases. Patient records were reviewed for hypertension, hypercholesterolemia, type 1 and type 2 diabetes, smoking, and migraine subtype. Smoking was categorized as never smoker vs. current- or ex-smoker.

### Brain MRI data acquisition

For cases, MRI scans were conducted on a Philips Ingenia 3T scanner (Philips Medical Systems, Best, the Netherlands). Axial 3D T2-weighted sequence with TR (Repetition Time) of 2,500 ms (milliseconds), TE (Time Echo) of 250 ms, matrix of 352 × 352, and slice thickness of 1 mm (millimeters) was obtained. We also obtained a coronal 2D FLAIR (Fluid Attenuation Inversion Recovery) sequence with TR of 4,800, TI (Time Inversion) of 1,650 ms, TE of 285 ms, matrix of 352 × 352, and slice thickness of 3 mm, sagittal 3DT1 sequence with TR of 81 ms, TE of 3.7 ms, matrix of 320 × 320 and slice thickness of 1 mm was obtained as well as susceptibility-weighted sequence with TR of 20 ms, TE of 27 ms, matrix of 512 × 512 and slice thickness of 2 mm. These sequences were obtained to find and exclude any brain pathology. MR angiography using an axial Time-Of-Flight (TOF) sequence with TR of 23 ms, TE of 3.5 ms, matrix of 640 × 640, and slice thickness of 1.2 mm was performed to evaluate the arteries of the brains. TOF images were interpreted as such and also 3D reconstructions in two different planes were built and interpreted.

For controls, MRI scans were conducted with any available 1.5-3T scanners at our catchment area with a routine MRI protocol that includes the following sequences; T1- and T2-weighted sequences, susceptibility-weighted sequences, and FLAIR sequences. MRI scanner type and field strength for each control is presented in Supplementary Table 1.

### Brain imaging and analysis

For controls and cases, two neuroradiologists (each with more than 10 years of experience in neuroradiology), blinded to case–control status and clinical data, evaluated independently the number, the location, and the size of WMHs from the fluid-attenuated inversion recovery (FLAIR)-sequences and T2-weighted images. In addition, microbleeds and lacunes of presumed vascular origin were evaluated in the same blinded fashion. Conflicting interpretations between the two radiologists were resolved by the consensus of the two interpreters.

Lesions ≥2 mm were categorized as WMH. Modified Scheltens' visual rating scale was used to evaluate WMH burden (Scheltens et al., 1993; Young et al., 2008; Lou et al., 2010), because WMHs located in the basal ganglia or brainstem were excluded according to neuroimaging standards for WMHs (Wardlaw et al., 2013). Modified Scheltens' visual rating scale provides a scoring system for periventricular WMH (0–9 points) and deep white matter hyperintensities (0–24 points) (Supplementary Table 2) (Young et al., 2008; Lou et al., 2010). WMH located < 10 mm from the ventricles was categorized as periventricular WMH (DeCarli et al., 2005). Subcortical WMHs was categorized as deep WMHs.

In addition, SmartBrush^®^ (Smartbrush 2.0, Brainlab AG, Feldkirchen, Germany) segmentation tool was used for manual segmentation for WMH volume from FLAIR-sequences. SmartBrush^®^ is a semi-automatic program that is FDA-approved for tumor-outlining and allows also manual segmentation. SmartBrush^®^ has good usability; the accuracy is less dependent on clinical experience and accuracy is comparable with other segmentation tools (Rana et al., 2015; Porz et al., 2016).

Lacune of presumed vascular origin was categorized as a round or ovoid, subcortical, fluid-filled cavity of between 3 and 15 mm in diameter from T1-weighted, T2-weighted, and FLAIR sequences (Wardlaw et al., 2013). Location of lacune was defined by vascular territory (Wardlaw et al., 2013). Microbleeds were evaluated from susceptibility sequences and differentiated from spontaneous intracerebral hemorrhages with T1-weighted and T2-weighted or FLAIR sequences (Wardlaw et al., 2013).

### Statistical analysis

All analyses were performed using SPSS Statistics 27 (IBM Corp., Armonk, NY, USA).

Mean ages within cases and between cases and controls were compared with a two-sample *t*-test. Percentage distribution of total, deep, and periventricular Scheltens' score were compared between cases and controls by using Chi-square test and Fisher's exact test to evaluate total, deep and periventricular WMH burden. Chi-square and Fisher's exact test were also used to test the association of categorical variables with Scheltens' score. Scheltens' score was dichotomized to 0 vs. ≥1 to compare prevalence and risk factors for total WMHs, deep WMHs, and periventricular WMHs within cases and controls, and between cases and controls with chi-square and Fisher's exact test. In those with positive WMH findings (Scheltens' score ≥ 1), Scheltens' score values were compared between cases and controls by using Mann-Whitney *U*-test. To further evaluate WMH burden, Scheltens' score was dichotomized to ≥2 vs. < 2 and ≥3 vs. < 3 and binary logistic regression was used to evaluate risk factors (Kawasaki disease, hypertension, diabetes type 1 or type 2, current smoker/ex-smoker and hypercholesterolemia) for Scheltens' score. *P*-values < 0.05 were considered as statistically significant. Missing data for each variable were excluded from the analyses. WMH volumes between cases and controls were compared by using Mann–Whitney *U*-test.

Cohen's kappa (k) analysis was used to evaluate inter-observer agreement for the WMH prevalence at the first evaluation round. Kappa value between 0.00 and 0.20 was defined as a slight agreement, 0.21–0.40 fair agreement, 0.41–0.60 moderate agreement, 0.61–0.80 substantial agreement, and 0.81–1.00 almost perfect agreement (Landis and Koch, 1977).

## Results

Demographics and mean Scheltens' scores for cases and controls are presented in Table 1 and the study population in the flow chart Supplementary Figure 1.

**Table 1:** Demographics for 40 cases and 160 controls.

**Variable**	**Cases** **(*n* = 40)**	**Controls (*n* = 160)**	***p*-value**
Mean age at time of brain MRI, years (SD)	33.3 (3.8)	32.8 (4.0)	0.53
**Sex**, ***n*** **(%)**			
Men	25 (62.5)	100 (62.5)	1.0
Female	15 (37.5)	60 (37.5)	1.0
Hypertension, *n* (%)	2 (5)	15 (9.4)	0.53
Hypercholesterolemia, *n* (%)	0 (0)	11 (6.9)	0.13
Type 1 diabetes, *n* (%)	1 (2.5)	3 (1.9)	1.0
Type 2 diabetes, *n* (%)	1 (2.5)	16 (10)	0.20
Migraine with aura, *n* (%)	2 (5.0)	69 (43.1)	**< 0.001**
Migraine without aura, *n* (%)	2 (5.0)	91(56.9)	**< 0.001**
Never smoker, *n* (%)	19 (47.5)	66 (54.1)	0.58
Smoker/ex-smoker, *n* (%)	21 (52.5)	56 (45.9)	0.58
Missing data for smoking, *n*	0	38	
**Brain MRI findings**			
Prevalence of WMHs, *n (*%)	8 (20)	18 (11.3)	0.14
Prevalence of deep WMHs, *n* (%)	8 (20)	18 (11.3)	0.14
Prevalence of periventricular WMHs, *n* (%)	3 (7.5)	0 (0)	**0.008**
Total WMH volume (cm^3^), median (IQR)	0.26 (0.34)	0.065 (0.075)	**0.039**
Total Scheltens' Score, median (IQR)	4.0 (4.5)	1.0 (0)	**0.003**
Scheltens' score for deep WMH, median (IQR)	3.0 (3.0)	1.0 (0)	**0.003**
Scheltens' score for periventricular WMH, median (IQR)	0 (1.5)	0 (0)	**0.007**
Lacune of presumed vascular origin, *n (%)*	3 (7.5)	3 (1.9)	0.096
**Vascular territory for lacune**			
Posterior cerebral artery, *n (%)*	2 (5.0)	3 (1.9)	0.26
Middle cerebral artery, *n (%)*	1 (2.5)	0 (0)	0.20
Hemorrhage/microbleeds, *n (%)*	0 (0)	0 (0)	1.0
Cerebral artery stenosis *n (%)*	0 (0)	N/A	

Number in bold indicate statistically significant results (*p* < 0.05).

Mean age for cases (*n* = 40) and controls (*n* = 160) was 33.3 (SD, 3.8) years and 32.8 (SD, 4.0) years, *P* > 0.5. Of the cases and controls, 62.5% were men. KD was diagnosed at an average age of 3.9 years and the mean follow-up time (from KD diagnosis to brain MRI) was 29.5 years (SD, 4.3). Of the 160 controls, 147 had undergone brain MRI because of migraine-related symptoms (Supplementary Table 3).

### Cases vs. controls

**Figure 1:**
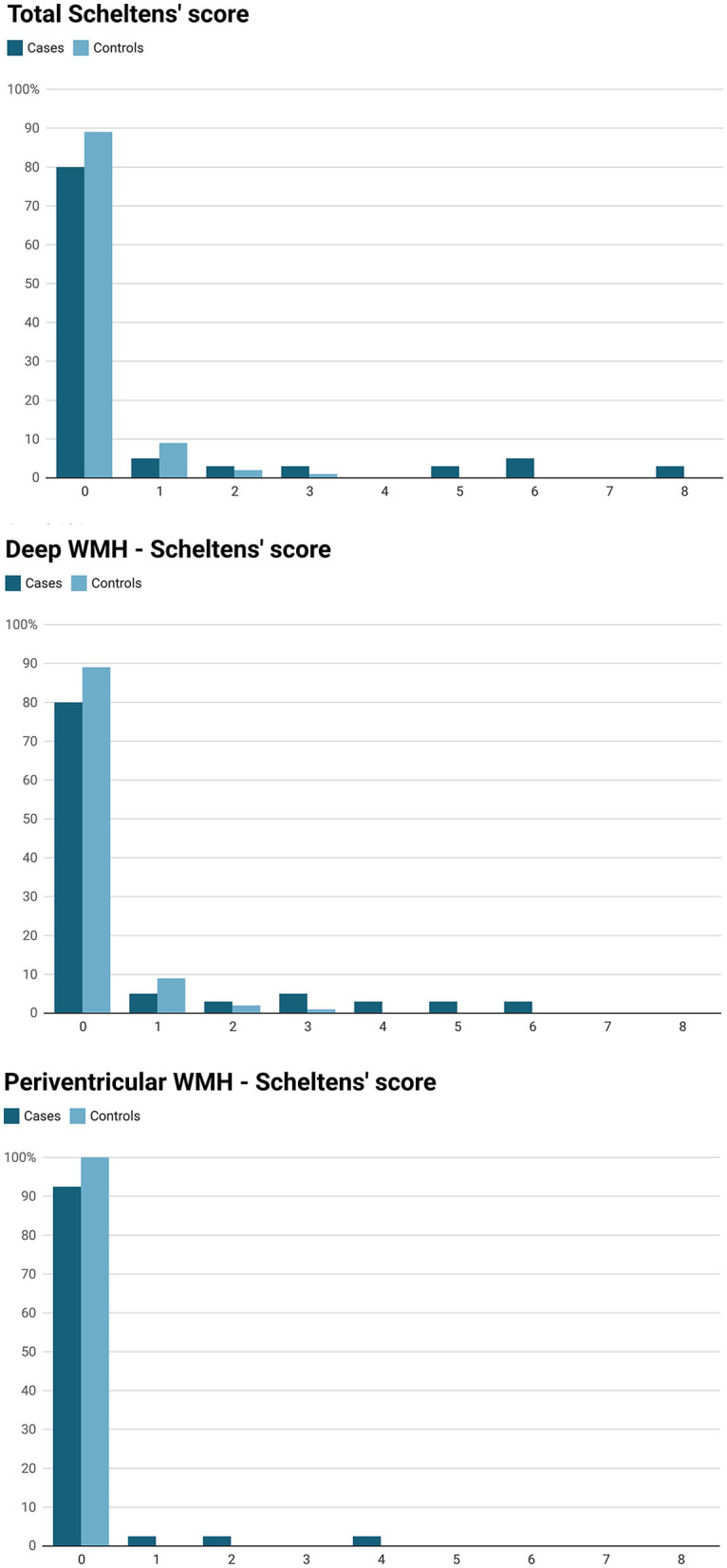
White matter hyperintensity scores (WMH) assesed by Scheltens' visual rating scale in 40 cases (Kawasaki disease) and 160 controls (migraine patients).

**Figure 2:**
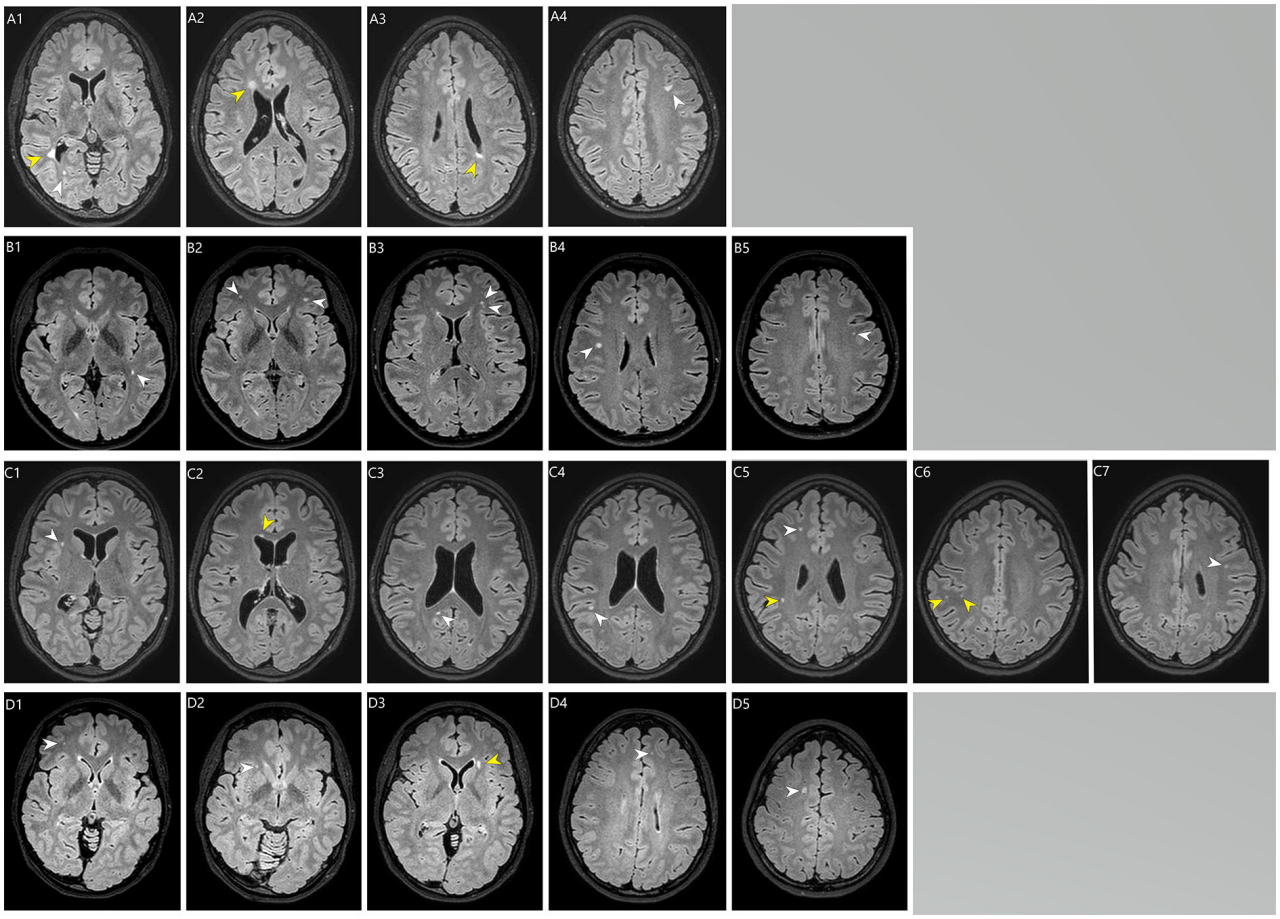
Brain MRI (T2-flair) of the white matter hyperintensities in four cases (Kawasaki disease). **(A1–A4)** a case (Kawasaki disease) with a total Scheltens' score of 8. **(A1)** Periventricular WMH > 5 mm in the right occipital horn (yellow arrow; Scheltens' score = 2) and in the deep occipital lobe WMH < 3 mm (white arrow; Scheltens' score = 1). **(A2)** Periventricular WMH > 5 mm in the right frontal horn (yellow arrow; Scheltens' score 2). **(A3)** Periventricular WMH > 5 mm in the left ventricle (yellow arrow; no score, because already score has been given from this area). **(A4)** deep WMH 4–10 mm in the left frontal lobe (white arrow; Scheltens' = score 3). **(B1–B4)** a case (Kawasaki disease) with a total Schelten score of 6. **(B1)** deep WMH 4–10 mm in the left temporal lobe (white arrow; Scheltens' score = 3). **(B2–B5)** multiple deep WMH in the both frontal lobes (white arrows), in the **(B2)** (left white arrow) and **(B4)** (white arrow) the size of the largest WMHs are 4–10 mm (Scheltens' score = 3). **(C1–C7)** a case (Kawasaki disease) with a total Schelten score of 6. **(C1,C5,C7)** multiple deep WMH < 3 mm in the frontal lobes (white arrows; Scheltens' score = 1). **(C2)** periventricular WMH ≤ 5 mm in the right frontal horn (yellow arrow; Scheltens' score = 1). **(C3)** deep WMH < 3mm in the right occipital lobe (white arrow; Scheltens' score = 1). **(C4)** (white arrow), **(C5)** (yellow arrow) and **(C6)** (yellow arrows): deep WMHs in the right parietal lobe, in the **(C4)** (white arrow) size of WMH is 4–10 mm (Scheltens' score = 1). **(D1–D5)** a case (Kawasaki disease) with a total Schelten score of 5. **(D1,D2,D4,D5)** Deep WMHs in the frontal lobes (white arrows). In the **(D5)** (white arrow) the size of the WMH is 4–10 mm (Scheltens' score = 3). **(D3)** periventricular WMH size > 5 mm next to left frontal horn (yellow arrow; Scheltens' score = 2).

Total WMH volume (median) in cases and controls was, 0.26 cm^3^ (IQR 0.34 cm^3^) and 0.065 cm^3^ (IQR 0.075 cm^3^), respectively, *P* = 0.039 (Table 1). According to Scheltens' score, cases had higher total WMH burden (*P* = 0.003), deep WMH burden (*P* = 0.003) and more periventricular WMHs (prevalence 7.5 vs. 0%, *P* = 0.008) compared to controls (Table 1). The distribution of Scheltens' score is presented separately in Figure 1. Brain MRI findings in cases with a highest Scheltens' scores are presented in Figure 2. Cases had greater risk of having total Scheltens' score ≥2 vs. < 2 (odds ratio, 6.88; 95% CI: 1.84–25.72, *P* = 0.0041) and ≥3 vs. < 3 (odds ratio, 22.71; 95% CI: 2.57–200.53, *P* = 0.0049) (Table 2). Lacune of presumed vascular origin was found in three cases (7.5%) and in three controls (1.9%), *P* = 0.096. There were no microbleeds in cases or in controls (Table 1).

**Table 2:** Risk factors total Scheltens' score ≥2 vs. < 2 and ≥3 vs. < 2 with binary logistic regression.

**Characteristic**	**Total Scheltens' score ≥2 vs. < 2**	**Total Scheltens' score ≥3 vs. < 2**
	**OR (95% CI), *p*-value**	**OR (95% CI), *p*-value**
Kawasaki disease	6.88 (1.84–25.72), ***p*** **=** **0.0041**	22.71 (2.57–200.53), ***p*** **=** **0.0049**
Diabetes mellitus (type 1 or 2)	0.94 (0.11–7.84), *p* = 0.96	–
Hypertension	1.29 (0.15–10.93), *p* = 0.81	2.39 (0.26–21.77), *p* = 0.44
Current smoker/ex–smoker	0.45 (0.11–1.81), *p* = 0.26	0.54 (0.10–3.03), *p* = 0.48
Hypercholesterolemia	–	–

Number in bold indicate statistically significant results (p < 0.05).

Hypercholesterolemia, hypertension, type 1 or type 2 diabetes, migraine with aura, or smoking were not significantly associated with the presentation of WMHs (*P* > 0.05) in cases (Supplementary Table 4) or in controls (Supplementary Table 5).

### Cases

None of the cases had been diagnosed with ischemic stroke during the time-period between KD diagnosis and follow-up MRI. Of the 37 cases with available information on complications during the acute onset of KD, seven had myocarditis during the acute phase of KD and two of them (28%) had periventricular WMHs, while those without myocarditis (*n* = 30) did not have any periventricular WMHs (0%), *P* = 0.03. Females had more deep (*P* = 0.002 and periventricular WMHs compared to males. Prevalence of deep WMHs was higher in patients with myocarditis (prevalence 42.9%) than in those without myocarditis (prevalence 13.3%), but the difference was not statistically significant (*P* = 0.1). Prevalence of deep and periventricular WMHs were similar in those who were treated with intravenous immunoglobulin and those who were not (*P* > 0.3). Five patients had coronary artery aneurysm or dilatation during the acute phase of KD and one of them had deep WMH. Two patients had coronary artery aneurysms and none of them had WMH findings (Table 2).

### Inter-observer agreement

Inter-observer agreement for WMHs was fair (*k* = 0.42, CI 95% 0.25–0.60) for the first evaluation. Of 200 study subjects, there were disagreements in 26 interpret of WMHs, which was resolved by consensus in the second evaluation. WMH volume and Scheltens' score had a good correlation ρ = 0.77, *p* < 0.0001.

## Discussion

In this long-term follow-up study of KD patients, WMH burden and prevalence of periventricular WMHs were significantly higher in patients with a history of KD compared to controls.

KD was first discovered in 1967, yet all long-term effects are still unknown (Cohen and Sundel, 2016). KD patients might have a higher risk for cardiovascular diseases and long-term effects on systemic arteries (McCrindle et al., 2017), but whether KD is linked to cerebrovascular diseases in the long-term is unclear (Muneuchi et al., 2006; Lin et al., 2022). To our knowledge, this study was the first to describe that history of KD is related to increased WMH burden in the long-term follow-up.

There are several possible mechanisms for why KD may be associated with an increased risk of WMHs. Hypoperfusion, the blood-brain barrier dysfunction, and inflammation are the potential underlying pathophysiological mechanisms for WMHs (Alber et al., 2019). Although KD is affecting predominantly medium-sized extracranial arteries, 1–30% might develop central nervous system symptoms (facial nerve paresis, meningeal irritation, bulging fontanelles, convulsions, somnolence, extreme irritability, headache) in acute KD (Tizard, 2005). Localized cerebral hypoperfusion has been reported in 29–72% of KD patients without neurological symptoms during the acute illness and lasting even several months afterward, possibly indicating cerebral vasculitis (Ichiyama et al., 1998; Hikita et al., 2011). Elevated inflammatory cytokines and pleocytosis in cerebrospinal fluid during the acute phase of KD has been found in 40–60% of patients, suggesting central nervous system inflammation in KD (Korematsu et al., 2007).

Interestingly, KD was particularly associated with periventricular WMHs (prevalence 7.5% in cases and 0% in controls). We also found that myocarditis during the acute phase of KD increased the risk for periventricular WMHs, one explanation for this finding could be hypoperfusion as well. Myocarditis is common in KD and can cause hemodynamic instability in severe cases (Dionne and Dahdah, 2018). Periventricular WMHs are often related to advanced age and cerebral small vessel disease and could be more susceptible to hypoperfusion (ten Dam et al., 2007). Periventricular WMHs and deep WMHs have different histopathological findings and clinical consequences, but studies suggest that periventricular and deep WMHs are probably a continuum of the same pathological process (Wardlaw et al., 2015). In KD patients, females had more periventricular and deep WMHs compared to males. One explanation could be that females may be more susceptible to WMHs due to genetic risk factors (Sachdev et al., 2016).

In our study, WMH burden was significantly higher in KD patients compared to controls with migraine, despite the fact that in previous studies migraine has been shown to be associated with an increased WMH burden compared to healthy controls (Kruit et al., 2004; Palm-Meinders et al., 2012; Hamedani et al., 2013). Prevalence of WMHs was 20% in KD patients, which is four times higher than reported in healthy young adults with a similar age (Hopkins et al., 2006). In contrast, the prevalence of WMH in cases (migraine patients) was comparable to pediatric migraine patients (11 vs. 11%) (Eidlitz-Markus et al., 2013), but lower than reported in young adults with migraine (Dobrynina et al., 2021).

KD is treated with intravenous immunoglobulin and aspirin to prevent coronary artery aneurysms (McCrindle et al., 2017). Intravenous immunoglobulin treatment may increase the risk for thromboembolic complications (Daniel et al., 2012; Ammann et al., 2016). In the present study, a relatively large proportion of KD patients were diagnosed before intravenous immunoglobulin treatment was established (Furusho et al., 1984), which allowed comparison of groups in terms of treatment. We did not find a significant difference in WMH prevalence or total WMH burden between KD patients treated with or without intravenous immunoglobulin. In KD, coronary artery aneurysms may predispose to more severe systemic inflammation (Lech et al., 2019). In our study, five patients had coronary artery dilatations or aneurysms and only one had periventricular and deep WMHs. Two KD patients had coronary artery aneurysms, but no WMHs. However, because of a small number of patients with coronary artery aneurysms, no conclusions can be drawn from this finding.

Increased WMH burden is associated with a higher risk for stroke, dementia, depression, and all-cause mortality (Au et al., 2006; Debette et al., 2019). Furthermore, increased WMH burden could be associated with different psychiatric disorders in children (Lyoo et al., 2002). Acute ischemic stroke after KD is uncommon and limited to single case reports (Wang et al., 2021). KD could be also associated with an increased risk of epilepsy and neurodevelopmental disorders, but the pathophysiology of these phenomena is unclear (Lin et al., 2019). We did not perform neuropsychological evaluation on study subjects, so no conclusions can be drawn on this issue from our study.

One of the limitations was the modest number of cases in this study. However, this was a population-based study as we reviewed all KD treated and diagnosed in our hospital district area and were able to recruit 40 of 60 patients who met the inclusion criteria for our study.

Migraine patients were selected as a control group because WMHs have been extensively studied in this population and migraine patients are a more homogenous population compared to headache patients in general which increases the repeatability of this study design. We acknowledge that migraine patients have an increased risk for WMHs (Dobrynina et al., 2021), but because we were able to show that WMHs burden is higher in KD compared to migraine patients this does not affect our conclusions and should strengthen our results. Another limitation of the control group was that they were selected retrospectively, which may have caused selection bias. However, one could assume that selection bias would rather have led to a higher number of WMH findings in control patients because migraine patients are not routinely undergoing brain MRI.

Another limitation is that we had no brain MRI imaging during the acute phase KD, so it is uncertain at which point WMHs occurred in Kawasaki disease patients.

There are limitations in the interpretation of WMHs. One major limitation was that brain imaging was performed on control patients with several different MRI scanners, which may have affected WMH interpretation. However, proposed image acquisition standards for WMH imaging (Wardlaw et al., 2013) were achieved also with controls. We evaluated WMH changes on a widely used quantitative visual rating scale (Wardlaw et al., 2013), one reason for this was that applying automatic segmentation tools to different scanners could have caused serious inter-scanner variability (Kuijf et al., 2019). Compared to automatic segmentation tools, visual rating scales are more prone to inter-rater variability, but on the other hand are more achievable methods (Wardlaw et al., 2013). Nevertheless, we used blinded review by two fellowship-trained neuroradiologists, and discrepancies were resolved using consensus. In addition, visual rating scales are comparable to automatic segmentation tools when assessing WMH burden (Valdés Hernández Mdel et al., 2013), but are inferior in grouping small differences and WMH progression (van den Heuvel et al., 2006).

## Conclusions

Our study suggests that patients with a history of Kawasaki disease might have an increased risk for WMHs, but it remains unclear whether WMHs occur during or after the acute phase of KD. More studies are needed to confirm our results.

## Data availability statement

The datasets generated during and/or analysed during the current study are available from the corresponding author on reasonable request.

## Ethics statement

The studies involving human participants were reviewed and approved by Ethics Committee of the Hospital District of Southwest Finland. The patients/participants (controls) provided their written informed consent to participate in this study. Informed consent was not required for controls, because controls were included from a retrospective register.

## Author contributions

DL helped conceptualize, develop and implement this study, analyzed and interpreted the data, wrote and edited the manuscript. RP and JH helped conceptualize this study, analyzed and interpreted the imaging data, and revised the manuscript for important intellectual content. PY and ES helped conceptualize this study, analyzed and interpreted the clinical data, and revised the manuscript for important intellectual content. TV helped conceptualize this, was responsible for statistical analysis, and revised the manuscript for important intellectual content. JK helped conceptualize this study, and revised the manuscript for important intellectual content. JR helped conceptualize and develop this study, and revised the manuscript for important intellectual content. MR helped conceptualize and develop this study, interpreted the clinical data, and revised the manuscript for important intellectual content. All authors approved the final manuscript as submitted and agree to be accountable for all aspects of the work.

## Funding

This study was supported by grant no. 17018 from the Pro Humanitate Foundation and a government research grant awarded to Turku University Hospital (DL).

## Conflict of interest

The authors declare that the research was conducted in the absence of any commercial or financial relationships that could be construed as a potential conflict of interest.

## Publisher's note

All claims expressed in this article are solely those of the authors and do not necessarily represent those of their affiliated organizations, or those of the publisher, the editors and the reviewers. Any product that may be evaluated in this article, or claim that may be made by its manufacturer, is not guaranteed or endorsed by the publisher.
