# Supplementary material for: Brain white matter hyperintensities in Kawasaki disease: A case–control study
Source: Front Neurosci. 2022 Oct 18;16:995480. doi: 10.3389/fnins.2022.995480 (PMC9623056; doi:10.3389/fnins.2022.995480)

Supplementary Material

# Supplementary Tables

**Supplementary Table 1**. MRI scanner types for each control and number of controls with white matter hyperintensities (WMH) for each MRI scanner.

| **MRI scanner** | **Field strenght (Tesla)** | **n=160** | **WMH (Scheltens’ score >0) (n=)** |
| --- | --- | --- | --- |
| Ingenia (Philips Healthcare) | 3 | 26 (16%) | 5 |
| Achieva (Philips) | 3 | 22 (14%) | 4 |
| Signa HDxt (GE healthcare) | 3 | 5 (3%) | 2 |
| MAGNETOM Skyra (Siemens Medical Systems, Erlangen, Germany) | 3 | 1 (1%) | 0 |
| Intera (Philips) | 1.5 | 28 (18%) | 2 |
| MAGNETOM Avanto (Siemens Medical Systems, Erlangen, Germany) | 1.5 | 24 (15%) | 0 |
| MAGNETOM Symphony (Siemens Medical Systems, Erlangen, Germany) | 1.5 | 13 (8%) | 1 |
| MAGNETOM Avanto fit (Siemens Medical Systems, Erlangen, Germany) | 1.5 | 11 (7%) | 1 |
| Genesis Signa (GE healthcare) | 1.5 | 8 (5%) | 0 |
| MAGNETOM Aera (Siemens Medical Systems, Erlangen, Germany) | 1.5 | 8 (5%) | 3 |
| Optima MR360 (GE healthcare) | 1.5 | 7 (4%) | 0 |
| MAGNETOM Essenza (Siemens Medical Systems, Erlangen, Germany) | 1.5 | 3 (2%) | 0 |
| Ingenia (Philips) | 1.5 | 3 (2%) | 0 |
| Gyroscan intera (Philips) | 1.5 | 1 (1%) | 0 |

**Supplementary Table 2.** Scheltens’ visual rating scale

| **Periventricular hyperintensities** | **Score (0-6)** |  |
| --- | --- | --- |
| Occipital caps | 0-2 | 0= absent |
| Frontal caps | 0-2 | 1= ≤5 mm |
| Bands along lateral ventricles | 0-2 | 2= > 5mm and < 10 mm |
|  |  |  |
| **White matter hyperintensities** | **Score (0-24)** |  |
| Frontal | 0-6 | 0= absent |
| Parietal | 0-6 | 1= <3 mm, n ≤5 |
| Occipital | 0-6 | 2= <3 mm, n > 6 |
| Temporal | 0-6 | 3= 4-10 mm, n ≤ 5 |
|  |  | 4= 4 mm, n > 6 |
|  |  | 5= > 11 mm, n > 1 |
|  |  | 6= confluent |

Semiquantative rating of white matter hyperintensities with the range of scale. In our study total Schelten score was the sum of the deep WMH and periventricular WMH Scheltens’ score.

Supplementary Table 3. Reason for brain MRI in 160 controls with migraine.

| **Symptom** | **Associated with migraine**  **(n=147)** | **Not associated with migraine**  **(n=13)** |
| --- | --- | --- |
|  |  |  |
| Frequent headache, n (%) | 70 (48) | 0 (0) |
| Sensory and/or motor symptoms, n (%) | 58 (39) | 2 (15) |
| Visual defects, n (%) | 16 (11) | 0 (0) |
| Cognitive symptoms, n (%) | 0 (0) | 3 (23) |
| Vertigo, n (%) | 3 (2) | 2 (15) |
| Aneurysm screening, n (%) | 0 (0) | 2 (15) |
| Seizure, n (%) | 0 (0) | 2 (15) |
| Abnormal tendon reflex, n (%) | 0 (0) | 1 (8) |
| Sleep walking, n (%) | 0 (0) | 1 (8) |

**Supplementary Table 4.** Clinical characteristics and their risk factors for deep and periventricular white matter hyperintensities (WMH) in 40 cases during the acute onset of Kawasaki disease (KD) and at the brain MRI.

| **Variables** | **Total**  **n=40** | **Deep WMH**  **n=8** | **p value** | **Periventricular WMH**  **n=3** | **p value** |
| --- | --- | --- | --- | --- | --- |
| **Acute onset of KD, demographics** |  |  |  |  |  |
| Age at KD diagnosis, mean years, (SD) | 3.9 (3.1) | Yes: 5.0 (3.9)  No: 3.7 (2.9) | 0.3 | Yes: 1.8 (1.2)  No: 4.1 (3.2) | 0.2 |
| **Sex, *n (%)*** |  |  |  |  |  |
| Female | 15 (37.5) | 7/15 (46.7) | **0.002** | 3/15 (20) | **0.046** |
| Men | 25 (62.5) | 1/25 (4.0) |  | 0/25 (0) |  |
| **IVIG treatment, n (%)** |  |  |  |  |  |
| Yes | 22 (52.5) | 5/22 (22.7) | 1.0 | 3/22 (13.6) | 0.3 |
| No | 15 (37.5) | 3/15 (20.0) |  | 0/15 (0) |  |
| Missing data | 3 (7.5) | 0/3 (0) |  | 0 (0/3) |  |
| **Coronary artery dilatation/aneurysm, n (%)** |  |  |  |  |  |
| Yes | 6 (15) | 1/6 (16.7) | 1.0 | 1/6 (16.7) | 0.3 |
| No | 31 (80) | 6/31 (19.4) |  | 1/31 (3.2) |  |
| Missing data | 3 (7.5) | 1/3 (33.3) |  | 1/3 (33.3) |  |
| **Coronary artery aneurysm, n (%)** |  |  |  |  |  |
| Yes | 2 (5.0) | 0/2 (0) | 1.0 | 0/2 (0) | 1.0 |
| No | 35 (87.5) | 7/35 (20) |  | 2/35 (5.7) |  |
| Missing data | 3 (7.5) | 1/3 (33.3) |  | 1/3 (33.3) |  |
| **Myocarditis, n (%)** |  |  |  |  |  |
| Yes | 7 (17.5) | 3/7 (42.9) | 0.1 | 2/7 (28.6) | **0.03** |
| No | 30 (75.0) | 4/30 (13.3) |  | 0/30 (0) |  |
| Missing data | 3 (7.5) | 1/3 (33.3) |  | 1/3 (33.3) |  |
| **Hepatitis, n (%)** |  |  |  |  |  |
| Yes | 5 (12.5) | 1/5 (20.0) | 1.00 | 0/5 (0) | 1.00 |
| No | 32 (80.0) | 6/32 (18.8) |  | 2/32 (6.3) |  |
| Missing data | 3 (7.5) | 1/3 (33.3) |  | 1/3 (33.3) |  |
| **Gallbladder hydrops, n (%)** |  |  |  |  |  |
| Yes | 2 (5.0) | 0/2 (0) | 1.00 | 0/2 (0) | 1.00 |
| No | 35 (87.5) | 7/35 (20) |  | 2/35 (5.7) |  |
| Missing data | 3 (7.5) | 1/3 (33.3) |  | 1/3 (33.3) |  |
| **Meningitis, n (%)** |  |  |  |  |  |
| Yes | 3 (7.5) | 1/3 (33.3) | 0.48 | 0/3 (0) | 1.00 |
| No | 34 (85.0) | 6/34 (17.7) |  | 2/34 (5.9) |  |
| Missing data | 3 (7.5) | 1/3 (33.3) |  | 1/3 (33.3) |  |
| **Anterior uveitis, n (%)** |  |  |  |  |  |
| Yes | 6 (15.0) | 0/6 (0) | 0.6 | 0/6 (0) | 1.0 |
| No | 31 (77.5) | 7/31 (22.6) |  | 2/31 (6.5) |  |
| Missing data | 3 (7.5) | 1/3 (33.3) |  | 1/3 (33.3) |  |
| **Relapse requiring re-treatment,** **n (%)** |  |  |  |  |  |
| Yes | 4 (10) | 1/4 (25) | 1.0 | 0/4 (0) | 1.0 |
| No | 36 (90) | 7/36 (19.4) |  | 3/36 (8.3) |  |
| **Kawasaki Disease Shock Syndrome** | 0 (0) | - |  | - |  |
| **Follow-up demographics** |  |  |  |  |  |
| Mean age at brain MRI, years (SD) | 33.3 (3.8) | Yes: 33.1 (2.9)  No: 33.3 (4.1) | 0.9 | Yes: 30.3 (2.9)  No: 33.5 (3.8) | 0.1 |
| **Migraine with aura, *n (%)*** |  |  |  |  |  |
| Yes | 2 (5) | 0/2 (0) | 1.0 | 0/2 (0) | 1.0 |
| No | 38 (95) | 8/38 (21.1) |  | 3/38 (7.9) |  |
| **Migraine without aura, *n (%)*** |  |  |  |  |  |
| Yes | 2 (5) | 0/2 (0) | 1.0 | 0/2 (0) | 1.0 |
| No | 38 (95) | 8/38 (21.1) |  | 3/38 (7.9) |  |
| **Hypertension, *n (%)*** |  |  |  |  |  |
| Yes | 1 (2.5) | 0/1 (0) | 1.0 | 0/1 (0) | 1.0 |
| No | 39 (97.5) | 8/39 (20.5) |  | 3/39 (7.7) |  |
| **Type 1 diabetes, *n (%)*** |  |  |  |  |  |
| Yes | 1 (2.5) | 0/1 (0) | 1.0 | 0/1 (0) | 1.0 |
| No | 0 (0) | 8/39 (20.5) |  | 3/39 (7.7) |  |
| **Type 2 diabetes, *n (%)*** | 0 (0) | - |  | - |  |
| **Hypercholesterolemia, *n (%)*** | 0 (0) | - |  | - |  |
| **Smoking, *n (%)*** |  |  |  |  |  |
| Smoker or ex-smoker | 21 (52.5) | 3/21 (14.3) | 0.4 | 1/21 (4.8) | 0.6 |
| Never smoker | 19 (47.5) | 5/19 (26.3) |  | 2/19 (10.5) |  |
| **Symptomatic ischemic stroke, *n (%)*** | 0 (0) | - |  | - | - |
| **Neurological symptoms, *n (%)*** | 0 (0) | - |  | - | - |
| **Depression, *n (%)*** |  |  |  |  |  |
| Yes | 3 (7.5) | 1/3 (33.3) | 0.5 | 1/3 (33.3) | 0.2 |
| No | 37 (92.5) | 7/37 (18.9) |  | 2/37 (5.4) |  |

Number in bold indicate statistically significant results (p<0.05).

**Supplementary Table 5.** Clinical characteristics and their risk factors for deep white matter hyperintensities (WMH) in 160 controls. There were no periventricular WMHs in controls.

| **Variables** | **Total n=160** | **Deep WMH**  **n=18** | **p value** |
| --- | --- | --- | --- |
| Mean age at brain MRI, years (SD) | 33.3 (3.8) | Yes: 34.4 (3.5)  No: 32.6 (4.1) | 0.3 |
| **Sex** |  |  |  |
| Female | 60 (37.5) | 5/60 (8.3) | 0.5 |
| Men | 100 (62.5) | 13/100 (13.0) |  |
| **Migraine, *n (%)*** |  |  |  |
| With aura | 69 (43.1) | 10/69 (14.5) | 0.3 |
| Without aura | 91 (56.9) | 8/91 (8.8) |  |
| **Hypertension, *n (%)*** |  |  |  |
| Yes | 15 (9.4) | 2/15 (13.3) | 0.68 |
| No | 145 (90.6) | 16/145 (11.0) |  |
| **Type 1 diabetes, *n (%)*** |  |  |  |
| Yes | 3 (1.9) | 0/3 (0) | 1.0 |
| No | 157 (98.2) | 18/157 (11.5) |  |
| **Type 2 diabetes, *n (%)*** |  | - |  |
| Yes | 16 (10) | 4/16 (25.0) | 0.086 |
| No | 144 (90) | 14/144 (9.7) |  |
| **Hypercholesterolemia, *n (%)*** |  | - |  |
| Yes | 11 (6.9) | 1/11 (9.1) | 1.0 |
| No | 149 (93.1) | 17/149 (11.4) |  |
| **Smoking**, ***n (%)*** |  |  |  |
| Smoker or ex-smoker | 56 (35.0) | 8/56 (14.3) | 0.4 |
| Never smoker | 66 (41.2) | 9/66 (13.6) |  |
| Missing data | 38 (23.8) | 1/38 (2.6) |  |
| **Symptomatic ischemic stroke, *n (%)*** | 0 (0) | - |  |

# Supplementary Figures

**Supplementary Figure 1**. Flow Chart


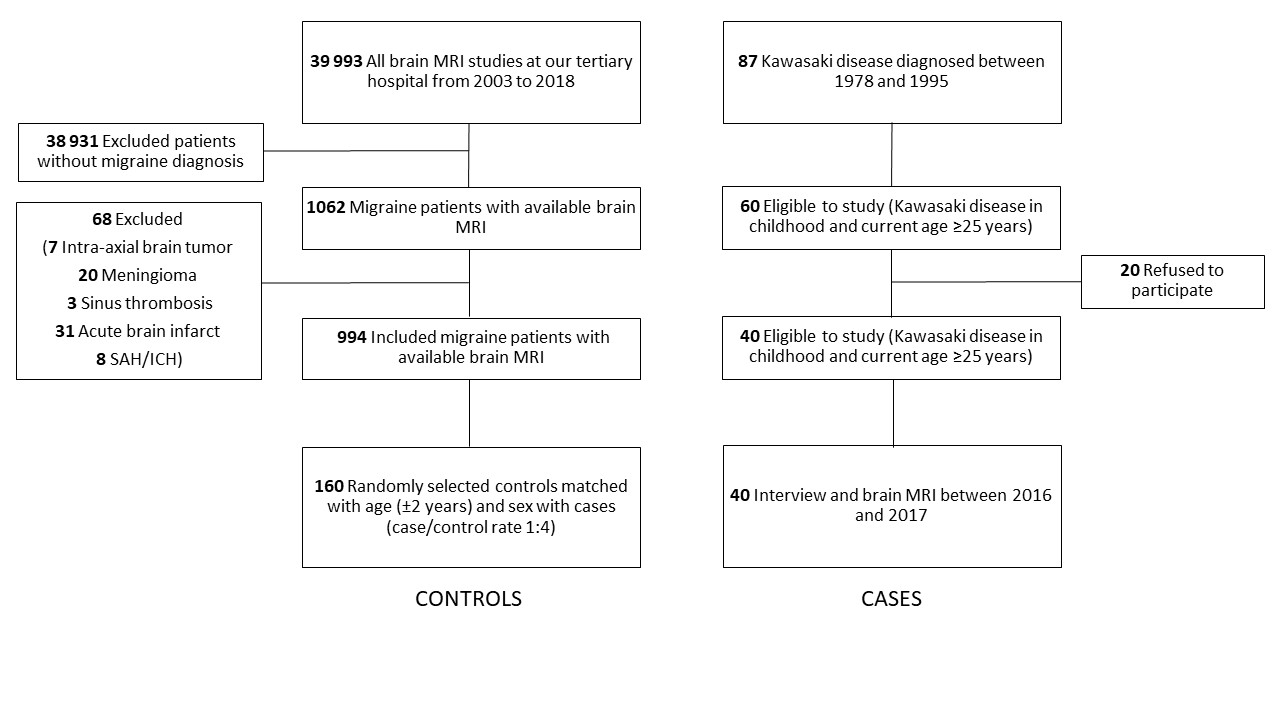

Supplement: Supplementary file 1 [file Table_1.docx]
